# Vildagliptin increases butyrate-producing bacteria in the gut of diabetic rats

**DOI:** 10.1371/journal.pone.0184735

**Published:** 2017-10-16

**Authors:** Qian Zhang, Xinhua Xiao, Ming Li, Miao Yu, Fan Ping, Jia Zheng, Tong Wang, Xiaojing Wang

**Affiliations:** Key Laboratory of Endocrinology, Translational Medicine Center, Ministry of Health, Department of Endocrinology, Peking Union Medical College Hospital, Peking Union Medical College, Chinese Academy of Medical Sciences, Beijing, China; East Tennessee State University, UNITED STATES

## Abstract

Emerging evidence supports a key role for the gut microbiota in metabolic diseases, including type 2 diabetes (T2D) and obesity. The dipeptidyl peptidase-4 inhibitor vildagliptin is highly efficacious in treating T2D. However, whether vildagliptin can alter the gut microbiome is still unclear. This study aimed to identify whether vildagliptin modifies the gut microbiota structure during T2D treatment. Diabetic Sprague-Dawley (SD) rats were induced by a high-fat diet and streptozotocin injection (HFD/STZ). Diabetic rats were orally administered a low dose of vildagliptin (LV, 0.01 g/kg/d vildagliptin), high dose of vildagliptin (HV, 0.02 g/kg/d vildagliptin), or normal saline for 12 weeks. Fasting blood glucose, blood glucose after glucose loading, and serum insulin levels were significantly reduced in the LV and HV groups compared with those in the T2D group. The serum GLP-1 level increased more in the vildagliptin-treated group than in the T2D group. Pyrosequencing of the V3-V4 regions of 16S rRNA genes revealed that vildagliptin significantly altered the gut microbiota. The operational taxonomic units (OTUs) and community richness (Chao1) index were significantly reduced in the vildagliptin and diabetic groups compared with those in the control group. At the phylum level, a higher relative abundance of *Bacteroidetes*, lower abundance of *Firmicutes*, and reduced ratio of *Fimicutes*/*Bacteroidetes* were observed in the vildagliptin-treated group. Moreover, vildagliptin treatment increased butyrate-producing bacteria, including *Baceroides* and *Erysipelotrichaeae*, in the diabetic rats. Moreover, *Lachnospira* abundance was significantly negatively correlated with fasting blood glucose levels. In conclusion, vildagliptin treatment could benefit the communities of the gut microbiota.

## Introduction

Type 2 diabetes (T2D) is rapidly increasing across the world. Environmental and genetic factors are considered the key underlying reasons for T2D epidemic. Tremendous attention and efforts have been given to decipher the “true story” of T2D. However, understanding how the location of candidate genes and single nucleotide polymorphisms (SNPs) identified in genome-wide association studies (GWAS) and the interaction between genes and the environment affect T2D is still limited. Increasing evidence supports a key role for the gut microbiota in the development of T2D [1, 2]. Previous studies have shown evidence that a high-fat diet changes the ratio of *Firmicutes* and *Bacteroidetes* phyla in the gut [3–5]. Mice fed with a high-fat and high-sugar diet showed a shift in the structure of the gut microbiota even within a single day [6]. More inspiring results came from germ- free animals. Several research groups have announced that germ free mice are resistant to diet-induced obesity and insulin resistance [7]. Transferring the fecal microbiota from ob/ob mice to germ-free mice results in increased body weight and insulin resistance [8, 9]. The following pathways are involved: insulin signaling [2], inflammation [10], glucose homeostasis [11], and increased glucagon-like peptide 1 (GLP-1) and peptide YY (PYY) secretion through short-chain fatty acids (SCFA) stimulation [12]. Therefore, Therapy that modulates the gut microbiota may prevent the growing T2D epidemic [13].

Vildagliptin, a dipeptidyl peptidase-4 inhibitor, was approved by the European Agency in 2008 for the treatment of T2D. This inhibitor avoids the enzymatic degradation of glucagon-like peptide 1 (GLP-1). GLP-1 is secreted from the intestinal L-cells. GLP-1 can stimulate insulin secretion from the pancreatic β-cells into the blood in response to glucose intake, reduced glucagon secretion from pancreatic α-cells, delayed gastric emptying and suppressed appetite, and it can protect pancreatic β-cells from apoptosis and promote β-cell proliferation [14].

Recently, an oral anti-diabetic agent, metformin, was proven to have beneficial effects on the gut microbiome in moderate hyperglycemia [15]. In a clinical trial, vildagliptin decreased the HbA1c and fasting blood glucose levels as an add-on therapy in patients with type 2 diabetes inadequately controlled by metformin [16]. Thus, we proposed our hypothesis that vildagliptin-treated diabetic rats also have beneficial modifications to their gut microbiome. However, studies about the effect of the whole gut microbiota in diabetic rats are limited. Therefore, we employed 454 pyrosequencing to explore the change in gut microbiota in vildagliptin-treated diabetic rats. The purpose of this research was to identify the important bacteria in the gut that could contribute to treating diabetes.

## Material and methods

### Generation of diabetic rats and drug treatment

This study was undertaken with the approval of the Animal Care Committee of the Peking Union Medical Hospital Animal Ethics Committee (Project XHDW-2015-0051, 15 Feb 2015), and all efforts were made to minimize suffering. Five-week-old male Sprague-Dawley (SD) rats (158.3 ± 14.8 g) were acquired from the Institute of Laboratory Animal Science, Chinese Academy of Medical Sciences and Peking Union Medical College (Beijing, China, SCXK-2014-0013). All rats were caged at 24 ± 1 ^o^C with lights on from 6:00 a.m. to 6:00 p.m.

The rats were fed a standard diet (kcal %: 10% fat, 20% protein, and 70% carbohydrate; 3.85 kcal/gm) or a high-fat diet (kcal %: 45% fat, 20% protein, and 35% carbohydrate; 4.73 kcal/gm, Research Diet, New Brunswick, NJ, USA). After exposure to the respective diets for 4 weeks, the rats were injected with STZ (30 mg/kg body weight, i.p.) or saline after being deprived of food for 10 h, and then, they continued with their original diet through the whole experimentation period. Fasting blood glucose (FBG) > 11.1 mmol/L was determined to be the standard definition for the type 2 diabetes model (HFD/STZ).

The diabetic rats were randomly divided into three subgroups: T2D treated with vehicle only (HFD/STZ, n = 6), T2D treated with 0.01 g vildagliptin/kg body weight/day (LV, n = 6), and T2D treated with 0.02 g vildagliptin/kg body weight/day (HFD/STZ+HV, n = 6). The normal diet-fed rats were orally administered vehicle only (NC, n = 6) or 0.02 g vildagliptin (NC+HV). After 12 weeks of treatment, fresh stools were collected by stimulating the anus in the NC, HFD/STZ, and HV group and immediately stored at -80°C for subsequent analysis. After 10 hours of food deprivation, the rats were anesthetized (ketamine 100 mg/kg i.p., Pharmacia and Upjohn Ltd., Crawley, UK), and then, the rats were sacrificed by decapitation. Blood samples were collected from the intraorbital retrobulbar plexus.

### Measurements of body weight, fasting blood glucose, and whole blood HbA1c

Body weight was recorded each month. Fasting blood glucose levels were determined with the Bayer Contour TS glucometer and blood glucose test strips (Bayer, Hamburg, Germany). Whole blood hemoglobin A1c (HbA1c) was measured by a high performance liquid chromatography (HPLC) method (Variant II, Bio-Rad Laboratories, Hercules, CA, USA).

### Oral glucose tolerance test (OGTT)

After a 12-week treatment, rats were fasted for 10 h before the glucose tolerance test. The oral glucose load was orally administered at 2 g / kg body weight. Tail blood glucose levels were measured at 30, 60 and 120 minutes after the glucose load. The area under the curve (AUC) was obtained by the linear trapezoid method [17].

### Measurement of serum insulin, GLP-1, IL-6, and HOMA-IR

Serum insulin, GLP-1 and IL-6 levels were determined by using an ELISA method (Millipore, Bellerica, MA, USA). The assessment of insulin resistance, the homeostasis model assessment of insulin resistance (HOMA-IR), was calculated from the following formula: FBG (mmol/L) x fasting serum insulin (μIU/mL) / 22.5.

### Fecal DNA extraction and 454 pyrophosphate sequencing

Total DNA was obtained from frozen feces using QIAamp DNA Stool Mini Kit (Qiagen, Hilden, Germany). The 16S rRNA genes were amplified using the 341F/806R primer set targeting the V3-V4 region (for 341F 5’-CCTAYGGGRBGCASCAG-3’; for 806R, 5’-GGACTACNNGGGTATCTAAT-3’). Purification of the amplified PCR products was conducted by using a QIAquick PCR purification kit (Qiagen, Hilden, Germany). Bacterial 16S rRNA pyrosequencing was performed on an Illumina HiSeq 2500 platform (Norcross, GA, USA).

After merging, the reads were analyzed by quality filtering, including removing sequences with an average quality score < 20 over a 50 bp sliding window, sequences shorter than 200 bp, sequences with homopolymers longer than six nucleotides, and sequences with ambiguous base calls or incorrect primer sequences. High quality reads were then annotated to operational taxonomic units (OTUs, 97% identity) using UPARSE version 7.1 [18], followed by the selection of representative sequences using QIIME software (version 1.7.0, Quantitative Insights into Microbial Ecology) [19]. OTUs were annotated with taxonomic information based on the RDP classifier version 2.2 [20] algorithm by using the GreenGene Database [21]. The relative abundance of each OTU was determined at the phylum, class, order, family, genus and species levels. Alpha (within a community) and beta diversity (between communities) were examined using QIIME software. For alpha diversity, Chao1 (community richness) and the Shannon-Wiener index (community diversity) were calculated. For beta diversity, principal coordinates analysis (PCoA) plots were determined by using both weighted and unweighted UniFrac [22]. In addition, linear discriminant analysis (LDA) of the effect size (LEfSe) was used to calculate OTU abundance and determine the differences among groups [23].

### Data analysis

The data are expressed as the means ± SD. When the data were normal and variances were equal, differences among the groups were analyzed using one-way ANOVA followed by Tukey’s post hoc test. Otherwise, a Kruskal-Wallis with Mann-Whitney test was applied. Spearman’s correlations between bacterial abundance and metabolic index were assessed. LEfSe was performed first based on the nonparametric factorial Kruskal-Wallis (KW) sum-rank test followed by the Wilcoxon Signed-Rank test. The threshold logarithmic LDA score was set at 3.0 and the scores were ranked. A *P*-value ≤ 0.05 was considered statistically significant. All data were analyzed with Prism version 5.0 for Windows (GraphPad Software, San Diego, CA, USA).

## Results

### Body weight

**Fig 1:**
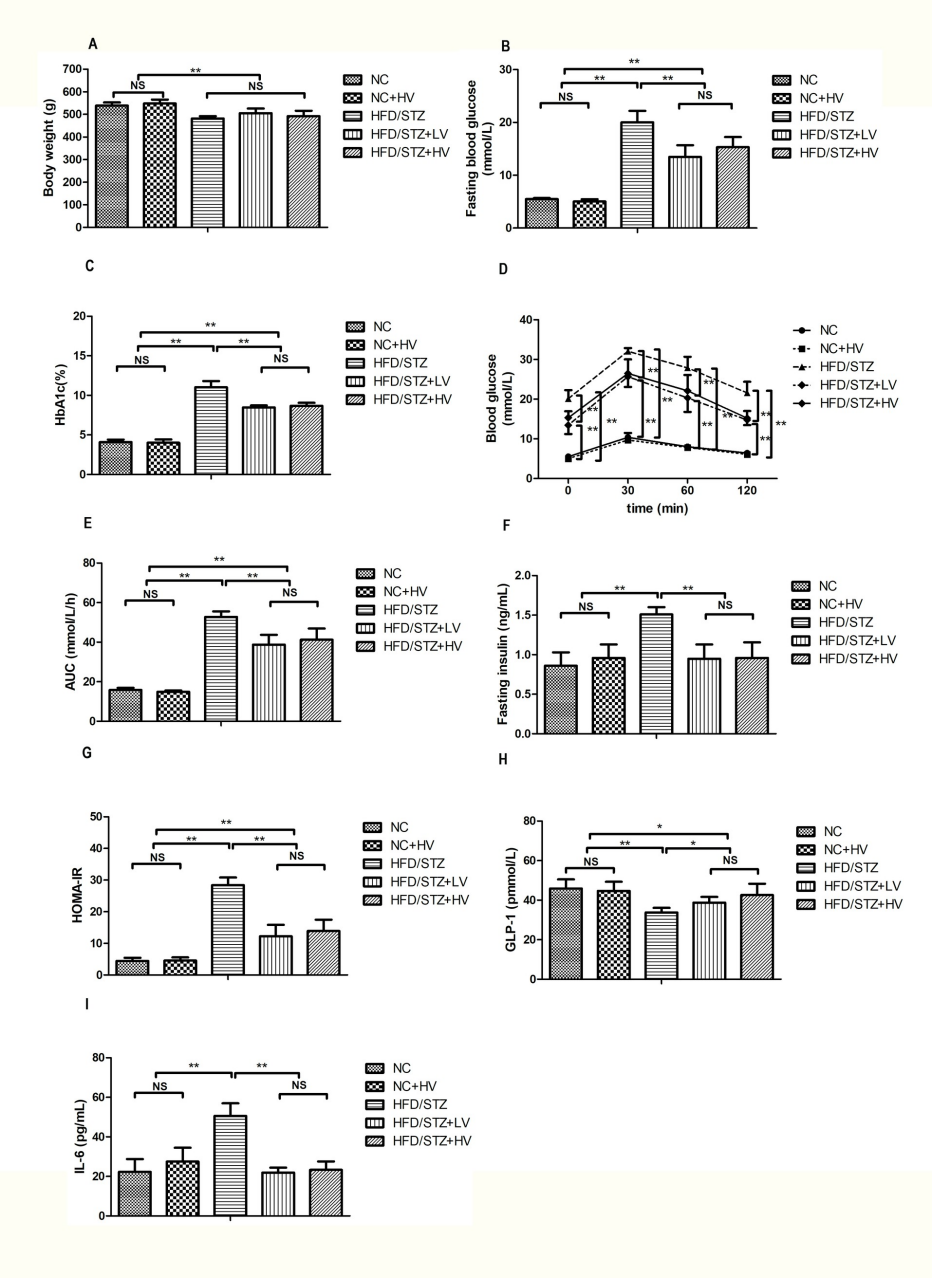
Effect of vildagliptin treatment on body weight, blood glucose, HbA1c, serum insulin, HOMA-IR, GLP-1, and IL-6. (A) body weight, (B) fasting blood glucose, (C) HbA1c, (D) glucose tolerance, (E) area under the curve of blood glucose, (F) serum insulin, (G) HOMA-IR, (H) GLP-1, (I) IL-6. Data was represented as mean ± SD. n = 6 in each group. * *P*<0.05, ** *P*<0.01, ^NS^ not significant.

The body weight of diabetic rats was slightly reduced compared with the normal controls (*P*<0.01, Fig 1A). The body weights of the diabetic rats and vildagliptin-treated rats did not significantly differ (Fig 1A).

### Fasting blood glucose, HbA1c and glucose tolerance

Fasting blood glucose and HbA1c levels in the HFD/STZ rats were significantly increased compared with those of the normal controls (*P*<0.01, Fig 1B). Both doses of vildagliptin treatment reduced the fasting blood glucose and HbA1c levels (*P*<0.01, Fig 1B and 1C). The blood glucose level after an oral glucose tolerance test was significantly higher in the diabetic group than in the normal controls (*P*<0.01, Fig 1D). Vildagliptin treatment reduced the blood glucose levels before and after glucose load, and the area under the curve of blood glucose (*P*<0.01, Fig 1D and 1E).

### Fasting insulin and homeostasis model assessment of insulin resistance (HOMA-IR), GLP-1, and IL-6

Serum insulin level, HOMA-IR, and IL-6 level in the diabetic rats were higher than that in the normal controls (*P*<0.01, Fig 1F, 1G and 1I), However, the serum GLP-1 level was lower in the diabetic rats than in the normal controls (*P*<0.01, Fig 1H) Vildagliptin reduced the serum insulin and IL-6 levels, alleviated insulin resistance, and increased serum GLP-1 in diabetic rats (*P*<0.05, Fig 1F, 1G, 1H and 1I).

### Global structural changes in the gut microbiota in response to vildagliptin treatment

**Fig 2:**
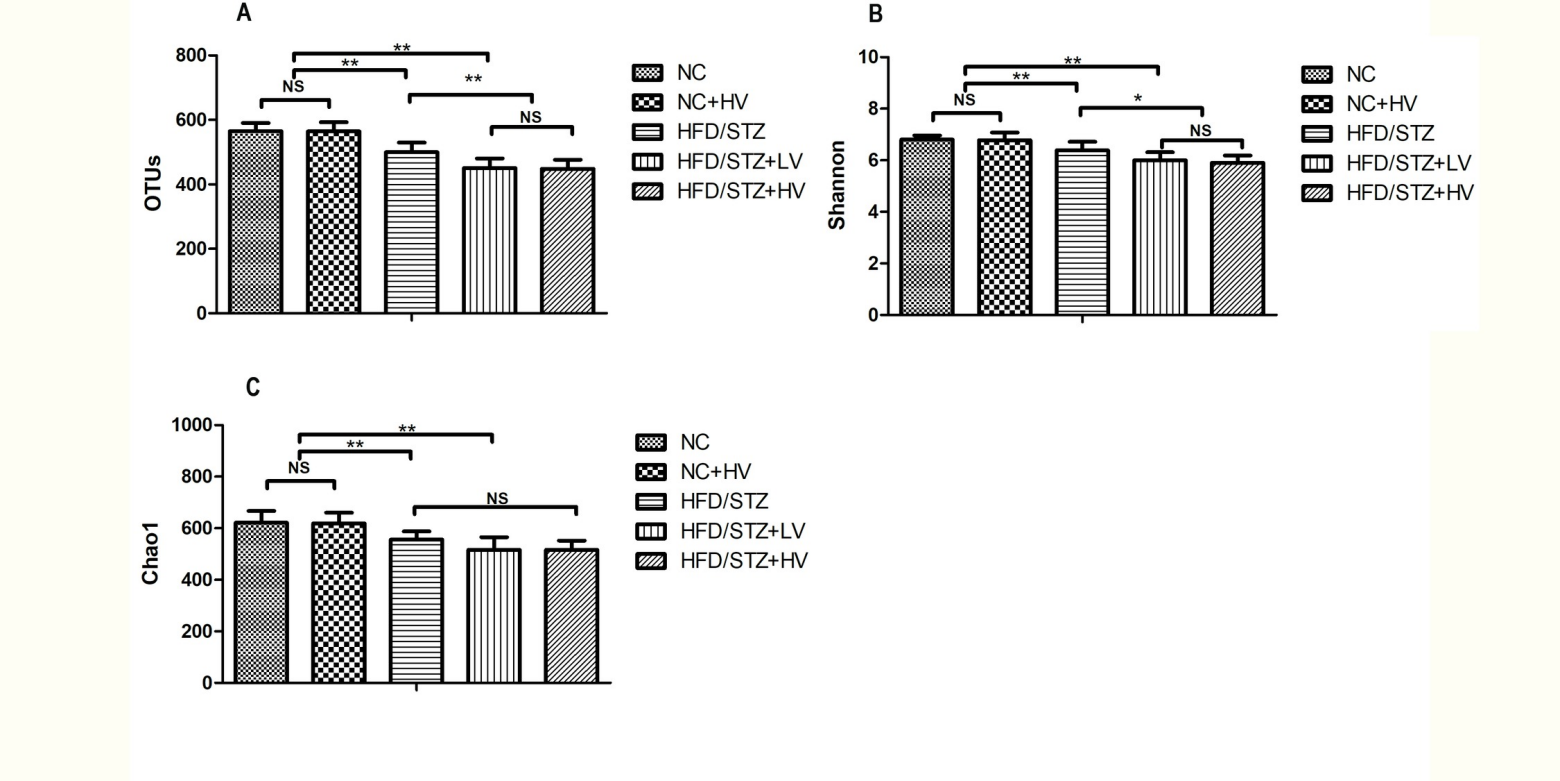
Pyrosequencing data summary. (A) number of OTUs, (B) Shannon index, and (C) Chao1 index. Data was represented as mean ± SD. n = 6 in each group. *P<0.05, ** *P*<0.01, ^NS^ not significant.

**Fig 3:**
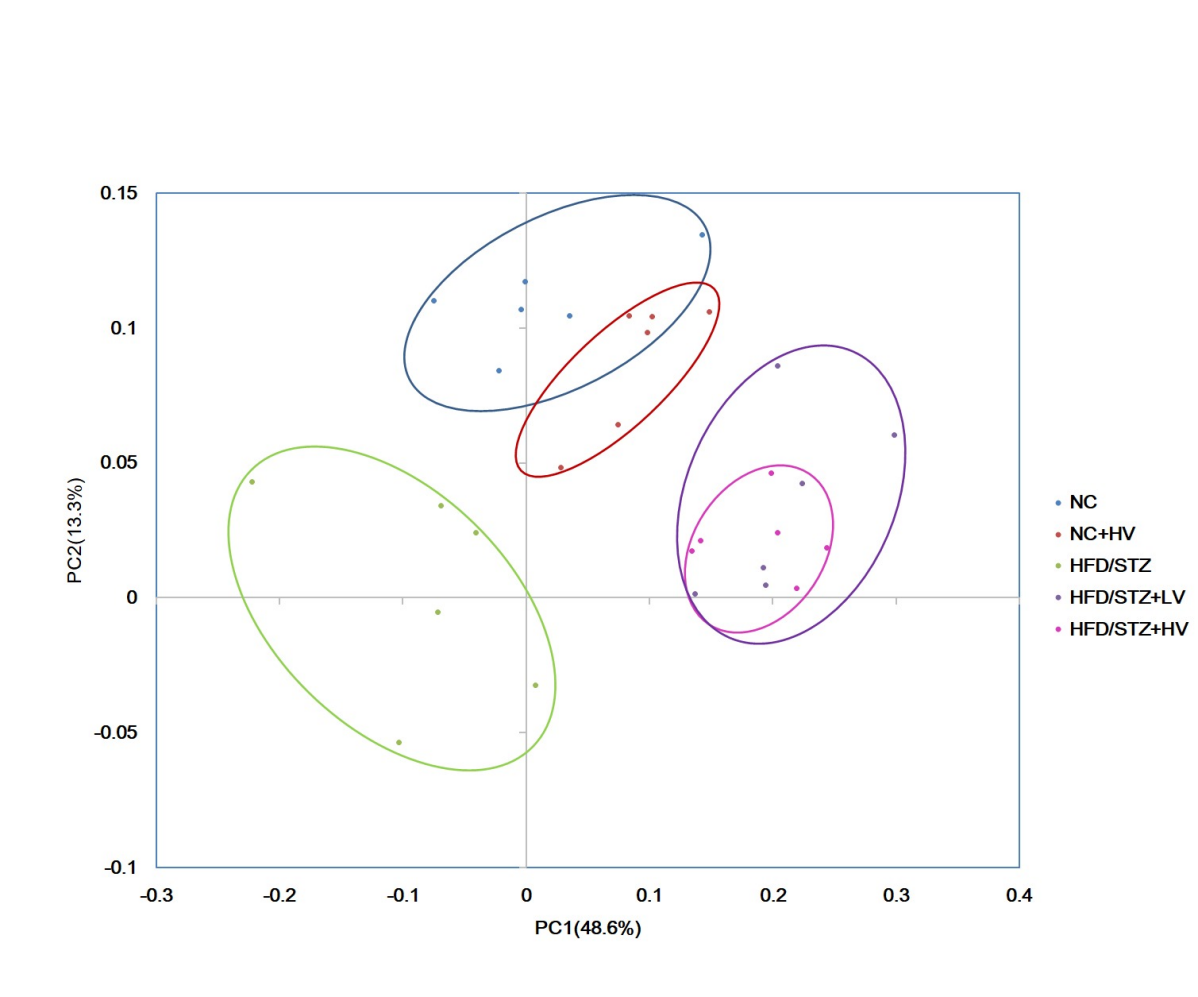
PCoA plots of weighted UniFrac distances of fecal microbial communities.

A total of 1,609,238 usable pyrosequencing reads (61,894 unique sequences) were obtained from 30 samples. The 16S sequence data generated in this study were submitted to the NCBI Sequence Read Archive (SRA) database (accession number SRP095711). After quality filtering, 1,289,798 reads (an average of 2213 sequences per sample) were identified as 918 OTUs at the 97% similarity level. Further statistical analysis of the estimators of OTUs and Shannon index showed that the HFD/STZ group had lower microbial diversity than the NC group (*P*<0.01, Fig 2A and 2B), and vildagliptin treatment reduced the microbial diversity in the HFD/STZ rats (*P*<0.05, Fig 2A and 2B). The Chao 1 index indicated that the gut microbiota richness in the HFD/STZ group was significantly lower than in the NC group (*P*<0.01, Fig 2C), however, there was similar richness among HFD/STZ, HFD/STZ+LV and HFD/STZ+HV groups (*P*>0.05, Fig 2C). Principal coordinates analysis of weighted UniFrac distances performed on the 97% OTU abundance matrix revealed a distinct separation in the beta diversity of the gut microbial communities among the NC, HFD/STZ, and HV groups. However, the NC and NC-HV groups shared some overlap (Fig 3). Alpha and beta diversity analyses of the NC+HV group showed that vildagliptin has little effect on the physiological state.

### The microbial structure of vildagliptin-treated rats differed substantially from that of diabetic rats

**Fig 4:**
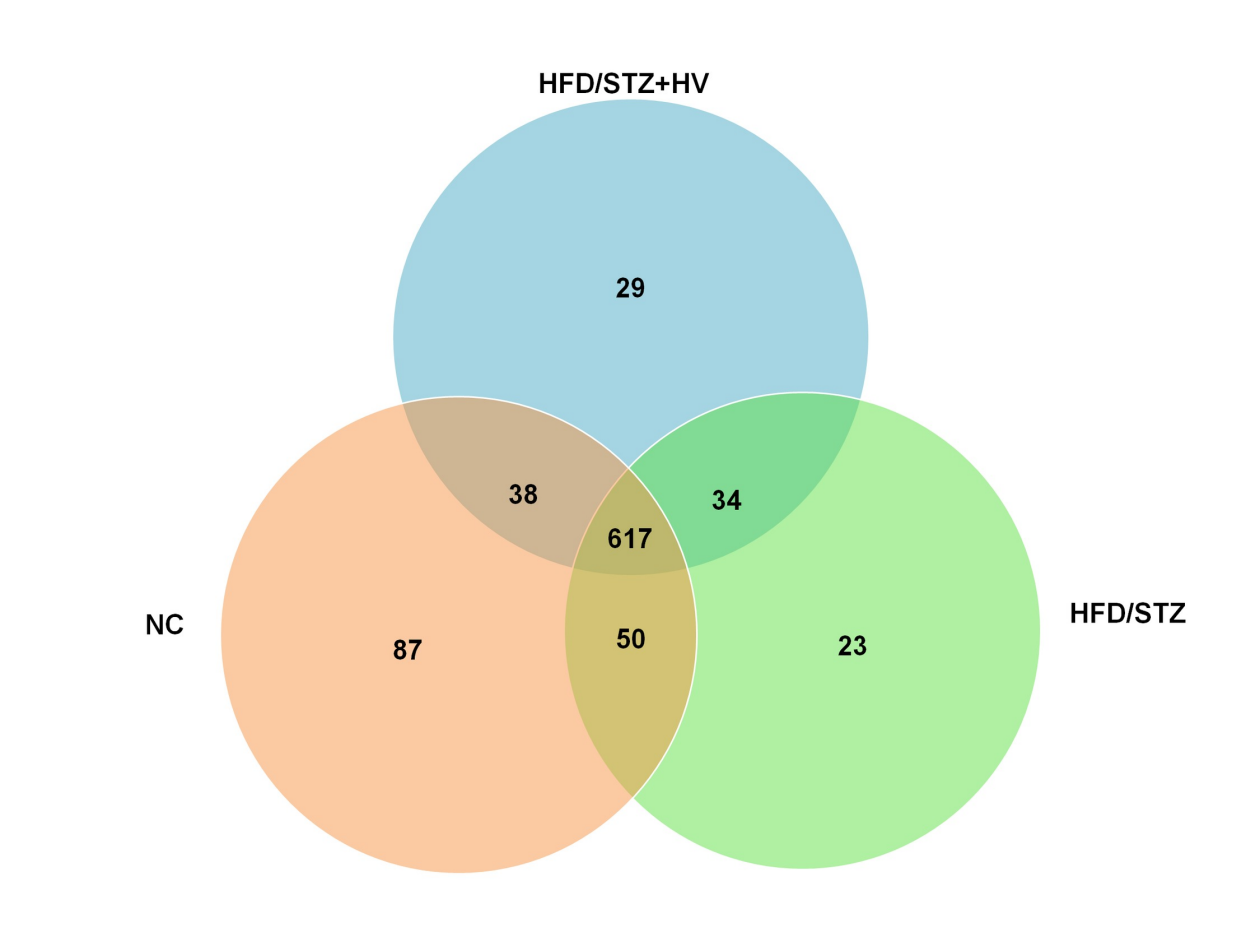
Shared OUT analysis of the different groups. Venn diagram showing the unique and shared OTUs (3% distance level) in the different groups.

To further explore the similarities and differences among normal, diabetic and vildagliptin-treated rats, we explored the similarities and distinctions in species distribution in the NC, HFD/STZ, and HFD/STZ+HV groups. As shown in Fig 4, we found that there were 617 shared species among the three groups, accounting for approximately three-fourths of the OTUs in each group. It is noteworthy that only 87 species were found in the NC group, 23 OTUs in the HFD/STZ group, and 29 OTUs in the HV group. At the genus level, the unique OTUs in the HV group were *Silanimonas* (from the *Xanthomonadaceae* family), *Coprobacillus* (from the *Erysipelotrichaceae* family), *Parabacteroides* (from the *Porphyromonadaceae* family), *Psychrosphaera* (from the *Pseudoalteromonadaceae* family), *Peptococcus* (from the *Peptococcaceae* family), *Anaerotruncus* (from the *Ruminococcaceae* family), *Gardnerella* (from the *Bifidobacteriaceae* family), *Ruminococcaceae_UCG-010* (from the *Ruminococcaceae* family), Acinetobacter (from the *Moraxellaceae* family), and *Alistipes* (from the *Rikenellaceae* family).

**Fig 5:**
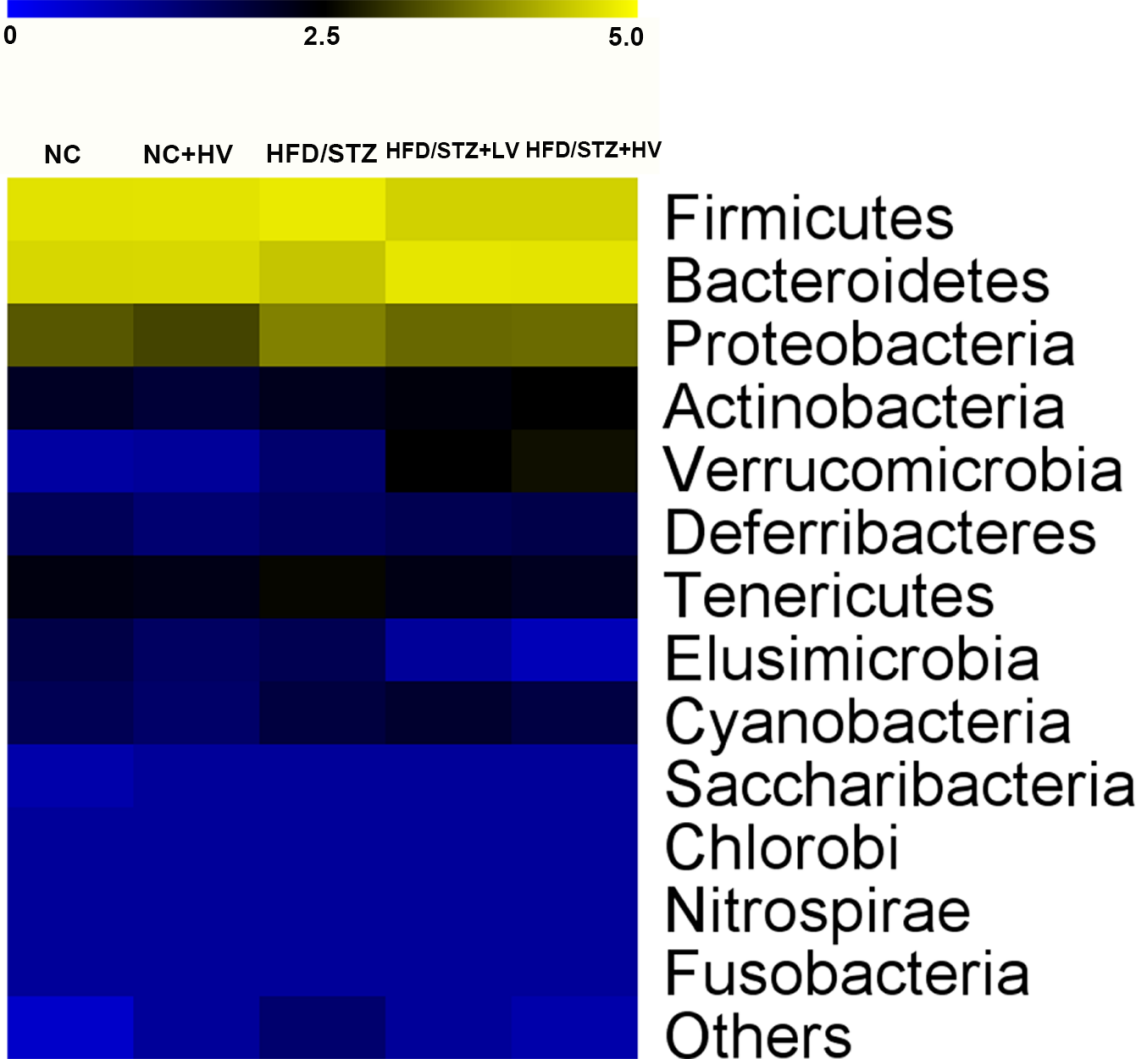
Heat map of the relative abundance (log_10_ transformation) of specific taxa in the fecal microbiota of rats in phyla level.

The microbiota in the HFD/STZ rats featured a high percentage of *Firmicutes* and both a low percentage of *Bacteroidetes* and a low ratio of *Bacteroidetes*: *Firmicutes* (*P*<0.01, S1 Table, Fig 5,). This composition was modified by vildagliptin treatment (*P*<0.01). Moreover, vildagliptin treatment reduced the relative abundance of *Tenericutes* and *Elusimicrobia* (*P*<0.05, S1 Table, Fig 5).

**Fig 6:**
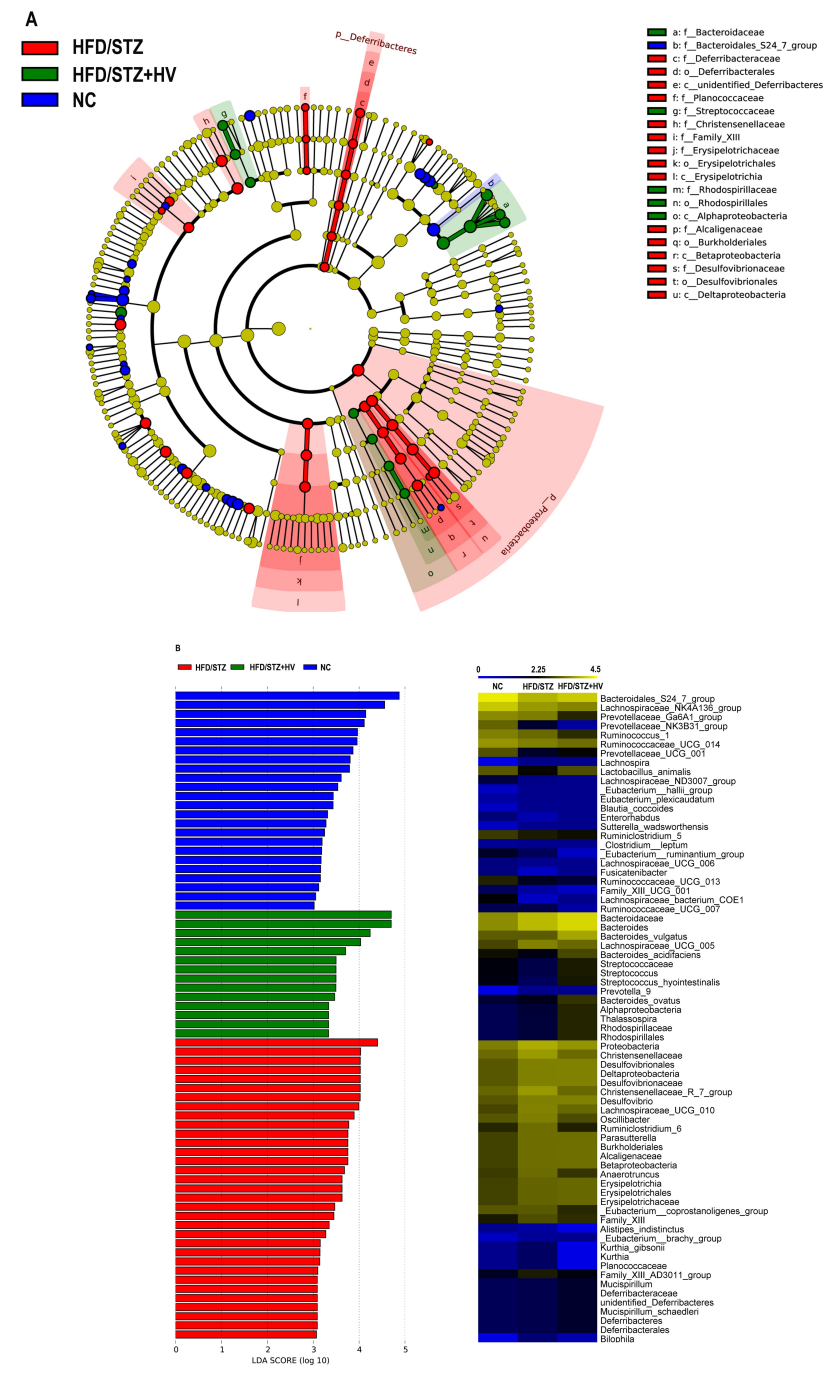
The LEfSe analysis of the gut microbiota in vildagliptin-treated rats differed significantly from that in NC and HFD/STZ group. (A) Differences are represented in the color of the most abundant group. (B) The left histogram shows the lineages with LDA values of 3.0 or higher as determined by LEfSe. The right heap map shows the relative abundance (log_10_ transformation) of OTUs.

There were significant alterations in the structure of intestinal bacteria species among the NC, HFD/STZ, and HFD/STZ+HV groups. A cladogram representation of the microbiota structure of these three conditions, the predominant bacteria in each condition performed by LEfSe, and the greatest differences in taxa among the three communities are presented in Fig 6 and S2 Table. We further discovered the key variables that separated the gut microbiota under vildagliptin treatment and identified 15 phylotypes as high-dimensional biomarkers. Four of these phylotypes were increased, and 11 phylotypes were decreased under vildagliptin treatment compared with the HFD/STZ group. The enriched phylotypes were the *Streptococcaceae* family (P<0.01), the genus *Streptococcus* within the family *Streptococcaceae* (*P*<0.01), the species *Bacteroides_acidifaciens* within the genera *Bacteroides* and the family *Bacteroidaceae* (*P*<0.01), and the species *Streptococcus_hyointestinalis* within the genus *Streptococcus* and the family *Streptococcaceae* (P<0.01). The decreased phylotypes were mainly the family *Ruminococcaceae*, such as the genera *Oscillibacter* (*P*<0.05), Ruminiclostridium_6 (*P*<0.05), *Anaerotruncus* (*P*<0.01), *Eubacterium_coprostanoligenes_group* (*P*<0.05), and *Ruminococcaceae_UCG_007* (*P*<0.05); the family *Planococcaceae*, such as the genus *Kurthia* (P<0.05), and the species *Kurthia_gibsonii* (*P*<0.05), the family *Christensenellaceae*, such as the genus *Christensenellaceae_R_7_group* (*P*<0.05), and the family *Prevotellaceae* (*P*<0.05).

### Identification of the key bacteria associated with the glucose metabolism index

**Fig 7:**
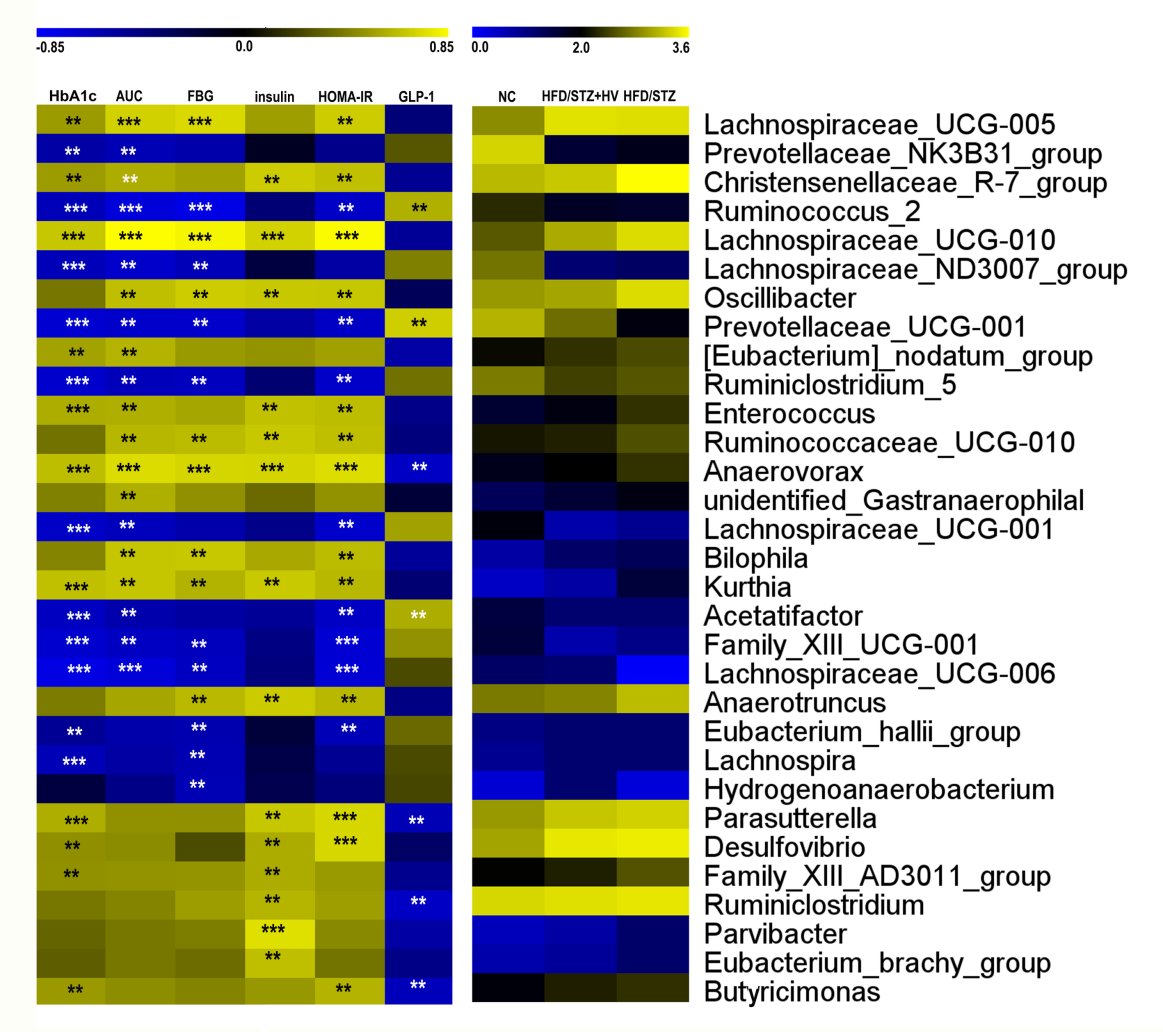
Correlation of the gut microbiota with glucose metabolism index. The left heat map shows the correlation coefficients between the OTUs and various glucose metabolism parameters. The right heat map shows the relative abundance (log10 transformed) of OTUs in the different groups. n = 6 in each group. **P<0.01, *** *P*<0.001.

The Spearman correlation of OTU counts (relative abundance) of bacterial genera with glucose metabolism index identified key OTUs that were potentially relevant to blood glucose control in different groups. There were nine genera associated with a decrease in blood glucose levels: *Ruminococcus_2*, *Lachnospiraceae_ND3007_group*, *Prevotellaceae_UCG-001*, *Ruminiclostridium_5*, *Family_XIII_UCG-006*, *Eubacterium_hallii_group*, *Lachnospira*, and *Hydrogenoanaerobacterium* (*P*<0.01). The genera associated with an increase in blood glucose levels were *Lachnospiraceae_UCG-005*, *Lachnospiraceae_UCG-010*, *Oscillibacter*, *Ruminococcaceae_UCG-010*, *Anaerovorax*, *Kurghia*, *Bilophila*, and *Anaerotruncus* (*P*<0.01, S3 Table, S4 Table, Fig 7). In addition, there were fourteen genera positively associated with fasting insulin levels: *Parasutterella*, *Desulfovibrio*, *Christensenellaceae_R-7_group*, *Lachnospiraceae_UCG-010*, *Oscillibacter*, *Anaerotruncus*, *Family_XIII_AD3011_group*, *Ruminococcaceae_UCG-010*, *Anaerovorax*, *Kurthia*, *Parvibacter*, and *Eubacterium_brachy_group*.

## Discussion

As expected, our study showed that vildagliptin diminished fasting blood glucose levels, and prevented both glucose intolerance and insulin resistance. In the gut microbiome analysis, we found significant changes among the control, diabetic and vildagliptin-treated groups. PCA revealed distinct fecal microbial communities among the NC, HFD/STZ, and HV groups. The HFD/STZ group had a lower microbial diversity and species richness compared with the NC group. Evidence shows that obesity is associated with reduced microbial diversity and richness [6, 24]. Dietary weight loss interventions have been shown to increase the gene richness of the microbiota [25]. In our study, the vildagliptin-treated rats displayed significantly reduced microbial diversity compared with that of the HFD/STZ rats. Metformin and berberine treatment also decreased the gut microbial diversity in a HFD-induced obesity rodent model [26, 27].

Our data showed that the rat gut microbiome was largely dominated by the phyla *Firmicutes* and *Bacteroidetes*. HFD/STZ induced diabetes was related to a significant shift in the gut microbiota, with a reduced percentage of *Bacteroidetes* and *Proteobacteria* and an increased proportion of the phyla *Firmicutes*. In addition, the *Bacteroidetes*/*Firmicutes* ratio also declined in the HFD/STZ group. Vildagliptin treatment normalized the *Bacteroidetes*/*Firmicutes* ratio, increased the abundance of the phylum *Bacteroidetes* and decreased the abundance of *Firmicutes*, *Tenericutes* (which lack a cell wall), and *Elusimicrobia*. Previous studies also address the ratio of the phyla *Bacteroidetes*/*Firmicutes* as an indicator of microbial imbalance related to a high-fat diet [8, 28, 29].

From the Venn diagram, we identified the unique family *Erysipelotrichaceae*, in vildagliptin treated rats. At the genus level, vildagliptin increased the level of *Bacteroides*. Moreover, *Lachnospira* was negatively correlated with FBG and HOMA-IR. *Erysipelotrichaceae*, *Bacteroides*, and *Lachnospira* are SCFA-producing taxa. Short chain fatty acids (SCFAs) are metabolic products of gut microbiota metabolism. The main products of SCFAs are acetate, propionate, and butyrate. SCFAs can stimulate host energy intake in the intestinal tract and is involved in the development of metabolic diseases [30–32]. Moreover, SCFAs could stimulate the secretion of GLP to affect the intestines [33, 34], and reduce gut permeability to inhibit serum lipopolysaccharide (LPS) and inflammatory cytokine levels [35, 36]. The abundance of the SCFA-producing bacteria, *Bacteroides*, in diabetic patients was reduced compared with subjects with normal glucose tolerance and with prediabetes [37, 38]. Moreover, physically fit subjects have an increased abundance of *Erysipelotrichaceae* in the gut microbiota [39]. Some oral antidiabetic agents, namely berberine and metformin, and energy-restricted diets could increase the SCFA-producing bacteria, *Bacteroides* and *Lachnospira*, in HF diet-induced obese rats and diabetic patients [40, 41].

Our data showed that *Prevotellaceae* abundance was lower in the vildagliptin treated rats than in the HFD/STZ rats. Brown and coworkers determined that the amount of *Prevotellaceae* in the gut microbiome was positively related to post-gestational diabetes mellitus (GDM) subjects. *Prevotella* are mucin-degrading bacteria that may be related to increased gut permeability [42]. Several studies report a negative relationship between the *Bacteroides*/*Prevotella* ratio and metabolic diseases, such as obesity [43] and non-alcoholic steatohepatitis [44].

Additionally, in our study, *Ruminococcaceae* abundance was lower in the vildagliptin-treated rats than in diabetic rats. In another study, there was a reduction in the abundance of *Ruminococcaceae* in rats treated with a bitter melon formulation (which could reduce fasting blood glucose) compared to the diabetic rats [45]. In addition, in our study, the abundance of *Oscillibacter*, one species within the taxa *Ruminococcaceae*, was reduced in vildagliptin-treated rats. In addition, *Oscillibacter* abundance was positively correlated with FBG, AUC in OGTT, fasting serum insulin, and HOMA-IR. *Oscillibacter* is positively correlated with gut permeability, which can affect gut barrier integrity [46]. The abundance of *Oscillibacter* increased under the HFD condition, and the abundance of these species declined after treatment with an inhibitor of the mTOR complex [47].

Our data also showed that the abundance of *Desulfovibrio* was positively correlated with fasting serum insulin level and HOMA-IR. As gram-negative bacteria, most members of *Desulfovibrio* are LPS producers [48, 49] and can damage the gut barrier [50]. A HFD can induce a leaky gut and increase bacterial lysis, allowing the LPS of gram-negative bacteria to enter the enterohepatic circulation [51]. LPS can activate the secretion of pro-inflammatory cytokines, leading to insulin resistance and relative metabolic disorders [35]. A previous study found that the family *Desulfovibrionaceae* was more common in IGT/obese rats, compared to normal controls [52]. Xie *et al*. found that *Desulfovibrio* was markedly increased in STZ-HFD-induced nonalcoholic steatohepatitis and was positively correlated with LPS levels [53].

Correlation analysis showed that *Bilophila* was positively correlated with FBG, AUC in OGTT and HOMA-IR. Recently, some studies have reported a positive relationship between the abundance of *Bilophila wadsworthia*, a high-fat diet and inflammation status [54, 55]. Our data also showed that vildagliptin could reduce the serum inflammatory cytokine IL-6 in diabetic rats.

## Conclusion

Our findings suggest that vildagliptin enriched SCFA-producing bacteria and reduced gut microbial diversity for improved gastrointestinal health and could eventually mediate their beneficial effects on the host, particularly in diabetes. The changes in the gut microbiota are associated with various metabolic biomarkers. We propose that modifying the specific structure and characteristics of the gut microbiota by vildagliptin treatment might become a therapeutic strategy for metabolic diseases, including T2D.

## Supporting information

S1 File The ARRIVE guidelines checklist. (PDF)

S1 Table The relative abundance (%) of specific taxa in phyla level. (DOCX)

S2 Table The relative abundance (%) of bacterial groups that showed statistical significance based on the LEfSe method. (DOCX)

S3 Table Correlation of the gut microbiota with glucose metabolism index. (DOCX)

S4 Table The relative abundance (%) of bacterial groups that showed statistical significance based on the correlation analysis of the gut microbiota with glucose metabolism index. (DOCX)
