# Supplementary material for: Vildagliptin increases butyrate-producing bacteria in the gut of diabetic rats
Source: PLoS One. 2017 Oct 16;12(10):e0184735. doi: 10.1371/journal.pone.0184735 (PMC5643055; doi:10.1371/journal.pone.0184735)
Supplement: S1 Table — (DOCX) [file pone.0184735.s002.docx]

**S1 Table. The relative abundance (%) of specific taxa in phyla level.**

| Taxonomy | NC | NC+HV | HFD/STZ | HFD/STZ+LV | HFD/STZ+HV |
| --- | --- | --- | --- | --- | --- |
| Firmicutes | 57.62(48.54-72.13) | 52.41(48.79-59.59) | 64.90(60.53-86.10)## | 36.57(30.45-38.46)**##$$ | 37.37(30.79-75.81)$$ |
| Bacteroidetes | 38.63(24.55-49.05) | 45.95(38.65-49.51) | 26.43(9.08-31.63)*## | 56.80(53.37-64.13)**##$$ | 55.81(15.75-64.35)$$ |
| Proteobacteria | 2.32(1.82-3.91) | 1.40(1.10-1.60)** | 5.95(3.56-14.33)*## | 5.07(3.85-8.34)**## | 3.70(3.18-9.04)*## |
| Actinobacteria | 0.14(0.08-0.26) | 0.05(0.03-0.10)** | 0.17(0.14-0.79) | 0.40(0.26-0.73)**## | 0.33(0.10-0.60) |
| Verrucomicrobia | 0.01(0.00-0.51) | 0.01(0.00-0.07) | 0.03(0.01-0.34) | 0.22(0.12-0.32)## | 0.46(0.00-0.94)# |
| Deferribacteres | 0.04(0.01-0.07) | 0.03(0.03-0.04) | 0.04(0.01-1.31) | 0.02(0.01-0.03)# | 0.06(0.04-0.10)## |
| Tenericutes | 0.22(0.06-0.35) | 0.20(0.14-0.25) | 0.38(0.14-0.83)# | 0.18(0.16-0.29) | 0.15(0.03-0.29)##$ |
| Elusimicrobia | 0.07(0.01-0.14) | 0.02(0.02-0.03) | 0.05(0.02-0.10) | 0.00(0.00-0.02)**##$$ | 0.01(0.00-0.01)*##$$ |
| Cyanobacteria | 0.05(0.02-0.08) | 0.01(0.00-0.02)** | 0.08(0.02-0.32) | 0.01(0.00-0.01)**$ | 0.07(0.01-0.20)# |
| Saccharibacteria | 0.01(0.00-0.06) | 0.00(0.00-0.00) | 0.00(0.00-0.01) | 0.00(0.00-0.00) | 0.00(0.00-0.00) |
| Chlorobi | 0.00(0.00-0.00) | 0.00(0.00-0.00) | 0.00(0.00-0.01) | 0.00(0.00-0.00) | 0.00(0.00-0.00) |
| Nitrospirae | 0.00(0.00-0.00) | 0.00(0.00-0.00) | 0.00(0.00-0.01) | 0.00(0.00-0.00) | 0.00(0.00-0.00) |
| Fusobacteria | 0.00(0.00-0.00) | 0.00(0.00-0.00) | 0.00(0.00-0.00) | 0.00(0.00-0.00) | 0.00(0.00-0.00) |
| Others | 0.00(0.00-0.01) | 0.00(0.00-0.01) | 0.03(0.01-0.05)# | 0.01(0.00-0.02) | 0.01(0.00-0.04) |
| Bacteroidetes/ Firmicutes | 0.67(0.34-1.01) | 0.88(0.65-1.01) | 0.40(0.11-0.50)*## | 1.57(1.39-2.11)**##$$ | 1.54(0.21-2.09)*$$ |

Data are presented as median (minimum-maximum), n=6 in each group. **P*<0.05, ***P*<0.01 versus NC; #*P*<0.05, ##*P*<0.01 versus NC+HV; $*P*<0.05, $$*P*<0.01, versus HFD/STZ.
