# Supplementary material for: Vildagliptin increases butyrate-producing bacteria in the gut of diabetic rats
Source: PLoS One. 2017 Oct 16;12(10):e0184735. doi: 10.1371/journal.pone.0184735 (PMC5643055; doi:10.1371/journal.pone.0184735)
Supplement: S2 Table — (DOCX) [file pone.0184735.s003.docx]

**S2 Table. The relative abundance (%) of bacterial groups that showed statistical significance based on the LEfSe method.**

| Bacterial groups | LDA | p-value | NC | | HFD/STZ | HFD/STZ+HV | |  |
| --- | --- | --- | --- | --- | --- | --- | --- | --- |
| Streptococcaceae | 3.497861 | 0.006475 | 0.063(0.013-0.247) | | 0.042(0.037-0.080) | 0.313(0.143-0.777)## | |  |
| _Eubacterium__hallii_group | 3.535445 | 0.004925 | 0.003(0.000-0.007) | | 0.000(0.000-0.000)* | 0.000(0.000-0.000)* | |  |
| Bacteroides | 4.699121 | 0.01154 | 2.460(0.880-5.790) | | 7.917(3.180-14.854) | 13.224(5.090-18.987)** | |  |
| Planococcaceae | 3.14217 | 0.004949 | 0.002(0.000-0.007) | | 0.023(0.003-0.073)** | 0.003(0.000-0.010)# | |  |
| Ruminococcus_1 | 3.965456 | 0.000986 | 2.422(1.997-3.357) | | 1.538(0.703-1.860) | 0.583(0.210-1.103)*** | |  |
| Deferribacterales | 3.088889 | 0.024448 | 0.030(0.007-0.073) | | 0.037(0.013-1.310) | 0.072(0.057-0.170)* | |  |
| Kurthia | 3.147733 | 0.004949 | 0.002(0.000-0.007) | | 0.023(0.003-0.073)** | 0.003(0.000-0.010)# | |  |
| Lachnospira | 3.804375 | 0.017554 | 0.002(0.000-0.017) | | 0.000(0.000-0.000) | 0.000(0.000-0.000)* | |  |
| Bacteroides_ovatus | 3.467427 | 0.005222 | 0.057(0.013-0.087) | | 0.107(0.013-0.190) | 0.178(0.120-1.590)** | |  |
| Desulfovibrionales | 4.026007 | 0.048668 | 1.065(0.517-1.570) | | 2.433(1.827-5.147)* | 3.103(0.533-6.034) | |  |
| _Eubacterium__ruminantium_group | 3.183927 | 0.016398 | 0.170(0.003-2.277) | | 0.028(0.007-0.087) | 0.008(0.000-0.060)* | |  |
| Lachnospiraceae_NK4A136_group | 4.55586 | 0.027424 | 15.774(4.263-28.661) | | 4.027(2.857-4.550) | 3.052(1.143-8.490)* | |  |
| Erysipelotrichaceae | 3.627993 | 0.03335 | 0.572(0.370-1.123) | | 1.373(0.867-4.183) | 1.588(0.993-3.123)* | |  |
| Fusicatenibacter | 3.162122 | 0.008193 | 0.010(0.000-0.013) | | 0.003(0.000-0.007) | 0.000(0.000-0.010)** | |  |
| Prevotellaceae_UCG_001 | 3.866072 | 0.004455 | 0.950(0.520-2.377) | | 0.077(0.007-0.153)** | 0.417(0.067-1.633) | |  |
| Oscillibacter | 3.891586 | 0.0079 | 1.042(0.337-1.317) | | 2.383(1.487-3.187)* | 1.183(0.317-2.447)# | |  |
| Streptococcus | 3.496403 | 0.006475 | 0.063(0.013-0.247) | | 0.040(0.037-0.080) | 0.313(0.140-0.777)## | |  |
| Lachnospiraceae_bacterium_COE1 | 3.053609 | 0.000532 | 0.152(0.043-0.697) | | 0.003(0.000-0.010)* | 0.000(0.000-0.023)*** | |  |
| Christensenellaceae | 4.032733 | 0.012336 | 1.202(0.727-2.690) | | 4.122(2.450-6.210)* | 1.573(0.470-4.703)# | |  |
| Prevotellaceae_NK3B31_group | 4.110597 | 0.003477 | 0.778(0.047-2.467) | | 0.067(0.003-0.113) | 0.033(0.003-0.123)** | |  |
| Deferribacteres | 3.088889 | 0.024448 | 0.030(0.007-0.073) | | 0.037(0.013-1.310) | 0.072(0.057-0.170)* | |  |
| Lactobacillus_animalis | 3.7904 | 0.040469 | 1.005(0.267-1.707) | | 0.212(0.030-0.683)* | 1.302(0.490-2.453) | |  |
| Eubacterium_plexicaudatum | 3.435769 | 0.004925 | 0.007(0.000-0.013) | | 0.000(0.000-0.000)* | 0.000(0.000-0.000)* | |  |
| _Eubacterium__coprostanoligenes_group | 3.470098 | 0.026834 | 0.888(0.440-1.370) | | 1.122(0.743-2.813) | 0.478(0.147-2.753)# | |  |
| Alistipes_indistinctus | 3.347446 | 0.028545 | 0.000(0.000-0.000) | | 0.007(0.000-0.010)* | 0.000(0.000-0.007) | |  |
| Alphaproteobacteria | 3.336823 | 0.002408 | 0.028(0.020-0.063) | | 0.058(0.030-0.230) | 0.187(0.063-1.797)** | |  |
| Rhodospirillaceae | 3.334486 | 0.004376 | 0.028(0.020-0.063) | | 0.053(0.030-0.230) | 0.187(0.060-1.793)** | |  |
| Family_XIII_UCG_001 | 3.120492 | 0.000544 | 0.035(0.010-0.050) | | 0.007(0.000-0.010)* | 0.003(0.000-0.007)*** | |  |
| Betaproteobacteria | 3.755426 | 0.019487 | 0.650(0.550-0.807) | | 1.723(1.293-4.280)* | 1.648(0.960-1.990) | |  |
| Prevotella_9 | 3.4938 | 0.031796 | 0.002(0.000-0.010) | | 0.010(0.007-0.013) | 0.007(0.000-0.027) | |  |
| Ruminococcaceae_UCG_013 | 3.156868 | 0.005758 | 0.378(0.153-0.730) | | 0.090(0.037-0.110) | 0.072(0.033-0.130)** | |  |
| Ruminococcaceae_UCG_014 | 3.954975 | 0.03143 | 4.072(1.823-5.510) | | 2.422(1.927-7.050) | 1.793(1.270-2.737)* | |  |
| Erysipelotrichales | 3.627993 | 0.03335 | 0.572(0.370-1.123) | | 1.373(0.867-4.183) | 1.588(0.993-3.123)* | |  |
| Mucispirillum_schaedleri | 3.088889 | 0.024448 | 0.030(0.007-0.073) | | 0.037(0.013-1.310) | 0.072(0.057-0.170)* | |  |
| Desulfovibrio | 4.023176 | 0.048668 | 1.062(0.517-1.563) | | 2.417(1.797-5.130)* | 3.088(0.530-6.004) | |  |
| Deltaproteobacteria | 4.026007 | 0.048668 | 1.065(0.517-1.570) | | 2.433(1.827-5.147)* | 3.103(0.533-6.034) | |  |
| Lachnospiraceae_UCG_010 | 3.995233 | 0.002028 | 0.757(0.537-0.893) | | 2.562(1.710-2.987)** | 1.697(0.813-8.317) | |  |
| Blautia_coccoides | 3.432551 | 0.017642 | 0.007(0.000-0.017) | | 0.000(0.000-0.000) | 0.000(0.000-0.000)* | |  |
| _Eubacterium__brachy_group | 3.274289 | 0.0317 | 0.003(0.000-0.007) | | 0.008(0.003-0.020) | 0.000(0.000-0.017) | |  |
| unidentified_Deferribacteres | 3.088889 | 0.024448 | 0.030(0.007-0.073) | | 0.037(0.013-1.310) | 0.072(0.057-0.170)* | |  |
| Family_XIII | 3.45007 | 0.010833 | 0.303(0.173-0.397) | | 0.903(0.520-1.293)** | 0.402(0.083-1.153) | |  |
| Bacteroidales_S24_7_group | 4.873253 | 0.022685 | 22.127(17.511-31.254) | | 7.100(4.260-10.880)* | 11.344(2.503-35.478) | |  |
| Ruminococcaceae_UCG_007 | 3.01999 | 0.003643 | 0.038(0.020-0.050) | | 0.037(0.010-0.043) | 0.007(0.000-0.013)**# | |  |
| Parasutterella | 3.756125 | 0.019531 | 0.647(0.543-0.807) | | 1.718(1.293-4.280)* | 1.640(0.957-1.990) | |  |
| Ruminiclostridium_6 | 3.774381 | 0.025553 | 0.387(0.253-0.917) | | 1.710(0.353-3.273) | 0.467(0.067-0.997)# | |  |
| Ruminiclostridium_5 | 3.247688 | 0.000546 | 0.570(0.513-0.747) | | 0.310(0.190-0.517)* | 0.260(0.223-0.410)*** | |  |
| Streptococcus_hyointestinalis | 3.496363 | 0.006972 | 0.052(0.010-0.240) | | 0.028(0.003-0.067) | 0.293(0.100-0.773)## | |  |
| Christensenellaceae_R_7_group | 4.025764 | 0.009775 | 1.107(0.640-2.543) | | 4.008(2.323-6.034)* | 1.508(0.447-4.523)* | |  |
| Family_XIII_AD3011_group | 3.100289 | 0.029525 | 0.092(0.047-0.137) | | 0.315(0.127-0.633)* | 0.125(0.017-0.357) | |  |
| Proteobacteria | 4.40304 | 0.001574 | 2.038(1.820-2.447) | | 5.952(3.557-14.327)** | 3.700(3.183-7.707) | |  |
| Bacteroidaceae | 4.699121 | 0.01154 | 2.460(0.880-5.790) | | 7.917(3.180-14.854) | 13.224(5.090-18.987)** | |  |
| Lachnospiraceae_ND3007_group | 3.60929 | 0.00281 | 0.048(0.033-0.097) | | 0.012(0.000-0.033)* | 0.003(0.000-0.047)** | |  |
| Thalassospira | 3.334486 | 0.004376 | 0.028(0.020-0.063) | | 0.053(0.030-0.230) | 0.187(0.060-1.793)** | |  |
| Erysipelotrichia | 3.627993 | 0.03335 | 0.572(0.370-1.123) | | 1.373(0.867-4.183) | 1.588(0.993-3.123)* | |  |
| Rhodospirillales | 3.334439 | 0.004376 | 0.028(0.020-0.063) | | 0.053(0.030-0.230) | 0.187(0.060-1.793)** | |  |
| Bilophila | 3.071007 | 0.03481 | 0.002(0.000-0.007) | | 0.017(0.003-0.030)* | 0.013(0.003-0.030) | |  |
| Deferribacteraceae | 3.088889 | 0.024448 | 0.030(0.007-0.073) | | 0.037(0.013-1.310) | 0.072(0.057-0.170)* | |  |
| Alcaligenaceae | 3.755426 | 0.019487 | 0.650(0.550-0.807) | | 1.723(1.293-4.280)* | 1.648(0.960-1.990) | |  |
| Kurthia_gibsonii | 3.153311 | 0.004949 | 0.002(0.000-0.007) | | 0.023(0.003-0.073)** | 0.003(0.000-0.010)# | |  |
| Lachnospiraceae_UCG_005 | 4.033138 | 0.005214 | 0.757(0.537-0.893) | | 2.562(1.710-2.987)** | 1.697(0.813-8.317) | |  |
| Lachnospiraceae_UCG_006 | 3.171077 | 0.00057 | 0.015(0.000-0.030) | | 0.000(0.000-0.003)* | 0.000(0.000-0.000)** | |  |
| Desulfovibrionaceae | 4.026007 | 0.048668 | 1.065(0.517-1.570) | | 2.433(1.827-5.147)* | 3.103(0.533-6.034) | |  |
| Bacteroides_vulgatus | 4.239864 | 0.048813 | 0.827(0.293-2.460) | | 1.170(0.913-2.650)* | 2.875(1.097-7.557) | |  |
| Prevotellaceae_Ga6A1_group | 4.14422 | 0.001664 | 4.102(1.967-6.680) | | 2.325(0.717-8.390) | 0.488(0.333-0.910)**# | |  |
| Enterorhabdus | 3.313605 | 0.00249 | 0.017(0.007-0.040) | | 0.005(0.003-0.017) | 0.002(0.000-0.007)** | |  |
| Sutterella_wadsworthensis | 3.278776 | 0.016544 | 0.003(0.000-0.010) | | 0.000(0.000-0.000)* | 0.000(0.000-0.003) | |  |
| Bacteroides_acidifaciens | 3.705292 | 0.002333 | 0.213(0.103-0.483) | | 0.125(0.023-0.337) | 0.768(0.480-2.943)## | |  |
| _Clostridium__leptum | 3.193268 | 0.00347 | 0.012(0.000-0.033) | | 0.000(0.000-0.000)** | 0.000(0.000-0.000)* | |  |
| Mucispirillum | 3.088889 | 0.024448 | 0.030(0.007-0.073) | | 0.037(0.013-1.310) | 0.072(0.057-0.170)* | |  |
| Burkholderiales | 3.755426 | 0.019487 | 0.650(0.550-0.807) | | 1.723(1.293-4.280)* | 1.648(0.960-1.990) | |  |
| Anaerotruncus | 3.680964 | 0.003183 | 0.612(0.400-0.790) | 1.618(0.997-1.980)** | | | 0.768(0.437-1.163)## | |

Data are presented as median (minimum-maximum), n=6 in each group. **P*<0.05, ***P*<0.01 versus NC，#*P*<0.05, ##*P*<0.01 versus HFD/STZ.
