# Supplementary material for: Vildagliptin increases butyrate-producing bacteria in the gut of diabetic rats
Source: PLoS One. 2017 Oct 16;12(10):e0184735. doi: 10.1371/journal.pone.0184735 (PMC5643055; doi:10.1371/journal.pone.0184735)
Supplement: S3 Table — (DOCX) [file pone.0184735.s004.docx]

**S3 Table. Correlation of the gut microbiota with glucose metabolism index**

| Bacterial group | HbA1c | | AUC | | FBG | | insulin | | HOMA-IR | | GLP-1 | |
| --- | --- | --- | --- | --- | --- | --- | --- | --- | --- | --- | --- | --- |
|  | Spearman r | P value | Spearman r | P value | Spearman r | P value | Spearman r | P value | Spearman r | P value | Spearman r | P value |
| Lachnospiraceae_UCG-005 | 0.5204 | 0.0032 | 0.7 | 0.0008 | 0.7275 | 0.0004 | 0.5316 | 0.0192 | 0.6845 | 0.0012 | -0.3977 | 0.0917 |
| Prevotellaceae_NK3B31_group | -0.55 | 0.0016 | -0.604 | 0.0062 | -0.569 | 0.011 | -0.1187 | 0.6284 | -0.4617 | 0.0466 | 0.2921 | 0.2249 |
| Christensenellaceae_R-7_group | 0.5246 | 0.0029 | 0.5807 | 0.0091 | 0.5432 | 0.0162 | 0.6842 | 0.0012 | 0.645 | 0.0029 | -0.4846 | 0.0355 |
| Ruminococcus_2 | -0.6173 | 0.0003 | -0.7205 | 0.0005 | -0.7647 | 0.0001 | -0.3888 | 0.1 | -0.6629 | 0.002 | 0.5903 | 0.0078 |
| Lachnospiraceae_UCG-010 | 0.6669 | < 0.0001 | 0.8491 | < 0.0001 | 0.8205 | < 0.0001 | 0.7053 | 0.0007 | 0.8407 | < 0.0001 | -0.5004 | 0.0291 |
| Lachnospiraceae_ND3007_group | -0.6171 | 0.0003 | -0.6797 | 0.0014 | -0.6171 | 0.0049 | -0.2189 | 0.368 | -0.5457 | 0.0157 | 0.4315 | 0.0651 |
| Oscillibacter | 0.3983 | 0.0293 | 0.6424 | 0.003 | 0.6866 | 0.0012 | 0.6705 | 0.0017 | 0.669 | 0.0017 | -0.2995 | 0.2128 |
| Prevotellaceae_UCG-001 | -0.695 | < 0.0001 | -0.6792 | 0.0014 | -0.6734 | 0.0016 | -0.545 | 0.0158 | -0.655 | 0.0023 | 0.6913 | 0.001 |
| [Eubacterium]_nodatum_group | 0.546 | 0.0018 | 0.6014 | 0.0065 | 0.5147 | 0.0241 | 0.4934 | 0.0318 | 0.5397 | 0.0171 | -0.5505 | 0.0146 |
| Ruminiclostridium_5 | -0.684 | < 0.0001 | -0.624 | 0.0043 | -0.6356 | 0.0034 | -0.3765 | 0.1121 | -0.6699 | 0.0017 | 0.383 | 0.1056 |
| Enterococcus | 0.5817 | 0.0007 | 0.5886 | 0.008 | 0.5588 | 0.0129 | 0.6485 | 0.0027 | 0.6333 | 0.0036 | -0.4577 | 0.0488 |
| Ruminococcaceae_UCG-010 | 0.3854 | 0.0355 | 0.6137 | 0.0052 | 0.6271 | 0.0041 | 0.6708 | 0.0017 | 0.6421 | 0.003 | -0.4134 | 0.0785 |
| Anaerovorax | 0.6385 | 0.0001 | 0.7367 | 0.0003 | 0.7137 | 0.0006 | 0.7103 | 0.0007 | 0.7423 | 0.0003 | -0.6551 | 0.0023 |
| unidentified_Gastranaerophilal | 0.4351 | 0.0163 | 0.5869 | 0.0082 | 0.4847 | 0.0354 | 0.3591 | 0.1311 | 0.4923 | 0.0323 | -0.1895 | 0.4373 |
| Lachnospiraceae_UCG-001 | -0.6652 | < 0.0001 | -0.6284 | 0.004 | -0.5703 | 0.0108 | -0.456 | 0.0497 | -0.6773 | 0.0014 | 0.5427 | 0.0164 |
| Bilophila | 0.4463 | 0.0134 | 0.6629 | 0.002 | 0.6716 | 0.0016 | 0.5635 | 0.012 | 0.6268 | 0.0041 | -0.5107 | 0.0255 |
| Kurthia | 0.6453 | 0.0001 | 0.6621 | 0.002 | 0.5998 | 0.0066 | 0.6751 | 0.0015 | 0.6194 | 0.0047 | -0.3809 | 0.1077 |
| Acetatifactor | -0.6462 | 0.0001 | -0.5777 | 0.0096 | -0.5354 | 0.0182 | -0.5053 | 0.0273 | -0.6599 | 0.0021 | 0.5825 | 0.0089 |
| Family_XIII_UCG-001 | -0.6883 | < 0.0001 | -0.6519 | 0.0025 | -0.6275 | 0.004 | -0.437 | 0.0614 | -0.7036 | 0.0008 | 0.4923 | 0.0323 |
| Lachnospiraceae_UCG-006 | -0.7589 | < 0.0001 | -0.7208 | 0.0005 | -0.6458 | 0.0028 | -0.4146 | 0.0776 | -0.7109 | 0.0006 | 0.2535 | 0.2949 |
| Anaerotruncus | 0.4172 | 0.0218 | 0.5579 | 0.0131 | 0.6222 | 0.0044 | 0.6807 | 0.0013 | 0.6169 | 0.0049 | -0.4346 | 0.063 |
| [Eubacterium]_hallii_group | -0.4963 | 0.0053 | -0.572 | 0.0105 | -0.5836 | 0.0087 | -0.199 | 0.4141 | -0.6097 | 0.0056 | 0.3538 | 0.1372 |
| Lachnospira | -0.6121 | 0.0003 | -0.5457 | 0.0156 | -0.5959 | 0.0071 | -0.2436 | 0.3149 | -0.4868 | 0.0345 | 0.2535 | 0.2949 |
| Hydrogenoanaerobacterium | -0.2166 | 0.2502 | -0.448 | 0.0544 | -0.6064 | 0.0059 | -0.2652 | 0.2724 | -0.4086 | 0.0824 | 0.2374 | 0.3278 |
| Parasutterella | 0.5855 | 0.0007 | 0.4895 | 0.0334 | 0.4835 | 0.036 | 0.6667 | 0.0018 | 0.7205 | 0.0005 | -0.6049 | 0.0061 |
| Desulfovibrio | 0.4818 | 0.007 | 0.4702 | 0.0422 | 0.2583 | 0.2856 | 0.5772 | 0.0097 | 0.7205 | 0.0005 | -0.3389 | 0.1558 |
| Family_XIII_AD3011_group | 0.4886 | 0.0061 | 0.4952 | 0.0311 | 0.4993 | 0.0295 | 0.5772 | 0.0093 | 0.5156 | 0.0239 | -0.4772 | 0.0388 |
| Ruminiclostridium | 0.4021 | 0.0276 | 0.4456 | 0.0559 | 0.5274 | 0.0203 | 0.607 | 0.0059 | 0.5309 | 0.0193 | -0.6558 | 0.0023 |
| Parvibacter | 0.3388 | 0.067 | 0.4005 | 0.0893 | 0.4203 | 0.0732 | 0.7499 | 0.0002 | 0.4627 | 0.0461 | -0.5454 | 0.0157 |
| [Eubacterium]_brachy_group | 0.3179 | 0.0869 | 0.4015 | 0.0884 | 0.3635 | 0.126 | 0.6412 | 0.0031 | 0.3876 | 0.1011 | -0.4528 | 0.0516 |
| Butyricimonas | 0.5171 | 0.0034 | 0.4754 | 0.0397 | 0.4414 | 0.0585 | 0.5088 | 0.0261 | 0.6029 | 0.0063 | -0.6225 | 0.0044 |
