# Supplementary material for: Vildagliptin increases butyrate-producing bacteria in the gut of diabetic rats
Source: PLoS One. 2017 Oct 16;12(10):e0184735. doi: 10.1371/journal.pone.0184735 (PMC5643055; doi:10.1371/journal.pone.0184735)
Supplement: S4 Table — (DOCX) [file pone.0184735.s005.docx]

**S4 Table. The relative abundance (%) of bacterial groups that showed statistical significance based on the correlation analysis of the gut microbiota with glucose metabolism index.**

| Parameter | NC | HFD/STZ | HFD/STZ+HV |
| --- | --- | --- | --- |
| Lachnospiraceae_UCG-005 | 0.757(0.537-0.893) | 2.562(1.710-2.987)** | 1.697(0.813-8.317) |
| Prevotellaceae_NK3B31_group | 0.778(0.047-2.467) | 0.067(0.003-0.113) | 0.033(0.003-0.123)** |
| Christensenellaceae_R-7_group | 1.107(0.640-2.543) | 4.008(2.323-6.034)* | 1.508(0.447-4.523)* |
| Ruminococcus_2 | 0.1117(0.0833-0.2567)** | 0.0233(0.0067-0.1000)* | 0.0433(0.0133-0.1333) |
| Lachnospiraceae_UCG-010 | 0.757(0.537-0.893) | 2.562(1.710-2.987)** | 1.697(0.813-8.317) |
| Lachnospiraceae_ND3007_group | 0.048(0.033-0.097) | 0.012(0.000-0.033)* | 0.003(0.000-0.047)** |
| Oscillibacter | 1.042(0.337-1.317) | 2.383(1.487-3.187)* | 1.183(0.317-2.447)# |
| Prevotellaceae_UCG-001 | 0.950(0.520-2.377) | 0.077(0.007-0.153)** | 0.417(0.067-1.633) |
| [Eubacterium]_nodatum_group | 0.0833(0.0333-0.1700)** | 0.2883(0.1600-0.4100) | 0.1600(0.0200-0.3133)# |
| Ruminiclostridium_5 | 0.570(0.513-0.747) | 0.310(0.190-0.517)* | 0.260(0.223-0.410)*** |
| Enterococcus | 0.0267(0.0100-0.0467)* | 0.2150(0.0300-0.4867) | 0.0367(0.0233-0.1500)# |
| Ruminococcaceae_UCG-010 | 0.0038(0.0015-0.0073)** | 0.0009(0.0004-0.0011)** | 0.0007(0.0003-0.0013) |
| Anaerovorax | 0.0450(0.0167-0.0933)** | 0.1967(0.1400-0.2933) | 0.0833(0.0267-0.1333)## |
| unidentified_Gastranaerophilal | 0.0133(0.0033-0.0367) | 0.0273(0.0167-0.2133) | 0.0217(0.0033-0.1300) |
| Lachnospiraceae_UCG-001 | 0.0633(0.0467-0.2100)* | 0.0083(0.0033-0.0467)** | 0.0017(0.0000-0.0133) |
| Bilophila | 0.002(0.000-0.007) | 0.017(0.003-0.030)* | 0.013(0.003-0.030) |
| Kurthia | 0.002(0.000-0.007) | 0.023(0.003-0.073)** | 0.003(0.000-0.010)# |
| Acetatifactor | 0.0350(0.0100-0.0567)** | 0.0000(0.0000-0.0000)** | 0.0000(0.0000-0.0000) |
| Family_XIII_UCG-001 | 0.0350(0.0100-0.0500)** | 0.0050(0.0000-0.0100)** | 0.0033(0.0000-0.0067) |
| Lachnospiraceae_UCG-006 | 0.0150(0.0000-0.0300)** | 0.0000(0.0000-0.0033)** | 0.0000(0.0000-0.0000) |
| Anaerotruncus | 0.612(0.400-0.790) | 1.618(0.997-1.980)** | 0.768(0.437-1.163)## |
| [Eubacterium]_hallii_group | 0.003(0.000-0.007) | 0.000(0.000-0.000)* | 0.000(0.000-0.000)* |
| Lachnospira | 0.002(0.000-0.017) | 0.000(0.000-0.000) | 0.000(0.000-0.000)* |
| Hydrogenoanaerobacterium | 0.0000(0.0000-0.0033) | 0.0000(0.0000-0.0000) | 0.0000(0.0000-0.0000) |
| Parasutterella | 0.647(0.543-0.807) | 1.718(1.293-4.280)* | 1.640(0.957-1.990) |
| Desulfovibrio | 1.062(0.517-1.563) | 2.417(1.797-5.130)* | 3.088(0.530-6.004) |
| Family_XIII_AD3011_group | 0.1500(0.0833-0.2033)** | 0.2783(0.1767-0.3533) | 0.1117(0.0333-0.2767)# |
| Ruminiclostridium | 0.0000(0.0000-0.0033)* | 0.0100(0.0000-0.0267) | 0.0000(0.0000-0.0100) |
| Parvibacter | 0.092(0.047-0.137) | 0.315(0.127-0.633)* | 0.125(0.017-0.357) |
| [Eubacterium]_brachy_group | 0.003(0.000-0.007) | 0.008(0.003-0.020) | 0.000(0.000-0.017) |
| Butyricimonas | 0.0417(0.0067-0.1067)** | 0.2167(0.0800-0.4267)* | 0.1183(0.0467-0.1867) |

Data are presented as median (minimum-maximum), n=6 in each group. **P*<0.05, ***P*<0.01 versus NC，#*P*<0.05, ##*P*<0.01 versus HFD/STZ.
